# Arterial Baroreflex Dysfunction Promotes Neuroinflammation by Activating the Platelet CD40L/Nuclear Factor Kappa B Signaling Pathway in Microglia and Astrocytes

**DOI:** 10.1007/s11064-022-03852-1

**Published:** 2023-01-02

**Authors:** Deping Kong, Rui Tan, Yongfeng Gao, Shan Gao, Zhaoyang Feng, Huibin Qi, Bowen Shen, Lili Yang, Xuri Shen, Xiuli Jing, Xiaomin Zhao

**Affiliations:** 1 Institute of Pharmacology, Shandong First Medical University & Shandong Academy of Medical Sciences, No. 619 Changcheng Road, 271016 Tai’an, People’s Republic of China; 2 School of Chemistry and Pharmaceutical Engineering, Shandong First Medical University & Shandong Academy of Medical Science, 271016 Tai’an, China

**Keywords:** Arterial baroreflex, Neuroinflammation, Astrocytes, Microglia, Platelet, CD40L

## Abstract

Arterial baroreflex (ABR) dysfunction has previously been associated with neuroinflammation, the most common pathological feature of neurological disorders. However, the mechanisms mediating ABR dysfunction-induced neuroinflammation are not fully understood. In the present study, we investigated the role of platelet CD40 ligand (CD40L) in neuroinflammation in an in vivo model of ABR dysfunction, and microglia and astrocyte activation in vitro. ABR dysfunction was induced in Sprague‒Dawley rats by sinoaortic denervation (SAD). We used ELSA and immunofluorescence to assess the effect of platelet CD40L on glial cell polarization and the secretion of inflammatory factors. By flow cytometry, we found that rats subjected to SAD showed a high level of platelet microaggregation and upregulation of CD40L on the platelet surface. The promotion of platelet invasion and accumulation was also observed in the brain tissues of rats subjected to SAD. In the animal model and cultured N9 microglia/C6 astrocytoma cells, platelet CD40L overexpression promoted neuroinflammation and activated M1 microglia, A1 astrocytes, and the nuclear factor kappa B (NFκB) signaling pathway. These effects were partially blocked by inhibiting platelet activity with clopidogrel or inhibiting CD40L-mediated signaling. Our results suggest that during ABR dysfunction, CD40L signaling in platelets converts microglia to the M1 phenotype and astrocytes to the A1 phenotype, activating NFκB and resulting in neuroinflammation. Thus, our study provides a novel understanding of the pathogenesis of ABR dysfunction-induced neuroinflammation and indicates that targeting platelet CD40L is beneficial for treating central nervous system (CNS) disorders associated with ABR dysfunction.

## Introduction

The arterial baroreflex (ABR) is an important physiological mechanism for blood pressure (BP) regulation, enabling the circulatory system to adapt to various conditions while maintaining BP and heart rate within a narrow normal range [1]. ABR function may be evaluated by assessing baroreflex sensitivity (BRS). Functional impairment of the ABR, an important feature of hypertension [2], has been reported to be a cardiovascular risk factor in disorders such as hypertension, diabetes, and obesity [3–6]. It has also been established that reduced ABR function can predict the risk of death after myocardial infarction and in heart failure and cerebrovascular diseases [7, 8]. Recently, more studies have revealed that ABR dysfunction is involved in central nervous system (CNS) disorders such as Parkinson’s disease [9], acute cerebral ischemia [10], major depressive disorder [11] and Alzheimer’s disease [12]. It is associated with worse nerve damage and shorter survival after stroke [13]. Neuroinflammation is a critical contributor to the pathophysiology of these neurological diseases [14–16].

An increasing amount of evidence suggests that ABR dysfunction can induce neuroinflammation. For example, it has been reported that, compared to rats with spontaneously high BRS, those with spontaneously low BRS show significantly increased levels of interleukin (IL)-1β and IL-6 in the brain [10]. Importantly, Liu et al. also observed that the levels of inflammatory factors are increased in the brains of rats with ABR dysfunction induced by sinoaortic denervation (SAD) [10]. In contrast, baroreflex activation through electrical aortic depressor nerve stimulation can attenuate LPS-induced changes in the levels of proinflammatory or anti-inflammatory cytokines in the hypothalamus. However, baroreflex activation cannot ameliorate the LPS-induced changes in the levels of all cytokines in peripheral tissues (the blood, spleen and heart) [17]. Additionally, hypertension, in which ABR function has been demonstrated to be reduced, can induce neuroinflammation [18]. Thus, ABR dysfunction may contribute to many CNS disorders by contributing to neuroinflammation [19]. To exploit this connection as a therapeutic target, studies need to elucidate how ABR dysfunction leads to neuroinflammation.

Platelets, as the smallest blood cells, participate not only in hemostasis but also in inflammation [20]. Platelets have also been linked to the pathogenesis of CNS disorders such as Alzheimer’s disease [21], cerebral malaria [22], and multiple sclerosis [23]. When platelets are activated, a high level of CD40 ligand (CD40L) is expressed on their surface, enabling them to interact with CD40-expressing cells such as microglia and astrocytes in the brain [24]. M1 microglia and A1 astrocytes are known to drive neuroinflammation [25, 26]. The M1/M2 balance and A1/A2 balance are dysregulated in the brain, and proinflammatory microglia and astrocytes oversecrete inflammatory factors (IL-1β, IL-6, tumor necrosis factor (TNF)-α), which are more conducive to neuroinflammation [27, 28]. Furthermore, the CD40L/CD40 dyad leads to the activation of nuclear factor kappa B (NFκB) [29], which is a marker of glial pathology under inflammatory conditions [30].

Therefore, we aimed to test the hypothesis that platelet CD40L is involved in ABR dysfunction-induced neuroinflammation and glial activation. Our study provides a new underlying mechanism by which ABR dysfunction causes neuroinflammation, promoting the development of novel treatments for related disorders.

## Materials and Methods

### Animals

Healthy male Sprague‒Dawley rats, weighing 220–250 g, were supplied by Jinan Pengyue Laboratory Animal Breeding (Jinan, China). The experimental procedures were approved by the Animal Care Ethics Committee of Shandong First Medical University.

### Animal Model of ABR Dysfunction

A rat model of ABR dysfunction was constructed using SAD as previously described [31]. Chronic SAD induces ABR dysfunction without causing hypertension [32]. In brief, the rats were anesthetized with pentobarbital (60 mg/kg), and then the aortic baroreceptor was denervated bilaterally by sectioning the aortic hypotensive nerves, cutting the superior laryngeal nerve near the vagus nerve, and removing the sections containing the superior cervical ganglion and sympathetic trunk. The carotid sinus baroreceptor was denervated bilaterally by dissecting the carotid bifurcation and its branches, followed by the application of 10% phenol to the external carotid, internal carotid, common carotid, and occipital arteries. SAD was considered complete when intravenous injection of phenylephrine (3–5 mg/kg) induced an increase in systolic blood pressure (SBP) of 50 mmHg and a change in heart rate of ≤ 20 beats/min. Sham-operated animals subjected to a midline neck incision followed by isolation of the bilateral neck muscles were used as controls.

In certain experiments, 4 weeks after SAD, the animals were administered clopidogrel (Jiangxi Revere Biotechnology, China) orally at a dose of 10 mg/kg/day for five weeks. The sham group was given the same quantity of vehicle by gavage.

### BP and BRS Measurement in Conscious Animals

As previously described [33], SBP, diastolic blood pressure (DBP), and heart period (HP) were continuously recorded. In brief, after the rats were anesthetized, a catheter (PE 50) was inserted into the left femoral vein for drug injection, while another catheter (PE 10) was placed in the lower abdominal aorta via the left femoral artery for BP measurement. Two days after catheter placement, BP and BRS (an index for assessment of ABR function) were determined in conscious, freely moving rats. The animals were given phenylephrine at a dose of 3–5 mg/kg to raise SBP by approximately 30–50 mmHg, and HP was continuously monitored while SBP returned to baseline. The slope of HP as a function of SBP was taken as the BRS (ms/mmHg) [31].

### Isolation of Rat Platelets

Whole blood was collected from the rat heart into anticoagulant-coated tubes containing sodium citrate (0.109 M) and centrifuged at 600 × g for 10 min. Platelet-rich plasma (PRP) was obtained by centrifugation at 600 × g for 10 min, and the concentration of the PRP was adjusted to 250 × 10^9^ platelets L^− 1^. This fraction was passed through a Sepharose 2B column (Solarbio, USA), and the concentration of the resulting gel-filtered platelets (GFPs) was adjusted to 160 × 10^9^ platelets L^− 1^ before use.

### Cell Culture

N9 murine microglia and C6 rat astrocytoma cells were obtained from Bluef Biotechnology (Shanghai, China). The cells were cultured in Dulbecco’s modified Eagle’s medium (DMEM; KeyGEN BioTECH, China) supplemented with 10% heat-inactivated fetal bovine serum at 37 ℃ in a humidified incubator (5% CO_2_). N9 or C6 cells were incubated with 50 µL of platelets (1.60 × 10^6^/mL) isolated from rats subjected to SAD or sham rats, clopidogrel (30 µM)- or vehicle (PBS)-pretreated platelets, or CD40L neutralizing antibody (30 µg/mL)- or vehicle (PBS)-pretreated platelets for 24 h.

### Platelet Microaggregation and CD40L Expression

To measure the rate of platelet microaggregation, platelets isolated from sham rats or rats subjected to SAD, were incubated with clopidogrel (30 µM) or vehicle (PBS) for 30 min. Then, the platelets were stimulated with 10 µM adenosine diphosphate (Sigma‒Aldrich, USA) or normal saline for 30 min. The platelets were stained with fluorescein (FITC)-conjugated mouse anti-rat CD61 antibody (BD Biosciences, USA), and platelet microaggregation was detected by a BD Accuri™ C6 Plus Flow Cytometer (BD Biosciences, USA).

To measure the surface expression of CD40L, platelets were stained with FITC-conjugated mouse anti-rat CD61 and phycoerythrin (PE)-conjugated mouse anti-rat CD40L antibodies (Santa Cruz Biotechnology, USA), and CD40L expression was measured by flow cytometry.

### Levels of Proinflammatory Cytokines

Levels of TNF-α and IL-6 in brain tissue lysates and cell culture supernatants were determined using commercial enzyme-linked immunosorbent assays for rat TNF-α and IL-6 (Shanghai Enzyme-linked Biotechnology, China) according to the manufacturer’s protocols.

### Immunofluorescence

Immunohistofluorescence analysis of the rat cortex and hippocampus was performed. After a continuous series of coronal brain Sect. (20 µm) were prepared using a freezing microtome (Leica, Germany), the sections were blocked using a mixture of 5% bovine serum albumin (BSA) and 0.1% Triton X-100 and then incubated overnight at 4 ℃ with the following primary antibodies: mouse anti-CD41 (1:50, Santa Cruz Biotechnology, USA), rabbit anti-glial fibrillatory acidic protein (GFAP; 1:250, Abcam, USA), mouse anti-GFAP (1:50, Abcam, USA), rabbit anti-ionized calcium-binding adaptor molecule 1 (Iba-1; 1:500, Abcam, USA), mouse anti-CD206 (1:50, Santa Cruz Biotechnology, USA), mouse anti-CD86 (1:100, Santa Cruz Biotechnology, USA), rabbit anti-complement component 3 (C3; 1:200, MyBioSource, USA), and mouse anti-S100 protein subtype a10 (S100a10; 1:200, Invitrogen, USA). Next, the slices were stained with Alexa Fluor 555-conjugated goat anti-rabbit and Alexa Fluor 488-conjugated donkey anti-mouse (1:500, Abcam, USA) for 1 h at room temperature. The nuclei were counterstained with 4′,6-diamidino-2-phenylindole (DAPI; Solarbio, China) for 5 min at room temperature. Images were taken on a fluorescence microscope (Axioscope 5, Zeiss, Germany), and the mean fluorescence intensity (MFI) and platelet count were quantified with ImageJ**-**win64 software (NIH, MD, USA).

Immunocytofluorescence of astrocytes and microglia was performed. After the cells were grown on coverslips, they were fixed with ice-cold 4% paraformaldehyde for 10 min. Subsequently, the cells were blocked with 1% BSA + 0.1% (v/v) Triton X-100 for 1 h at room temperature. Then, they were incubated with primary antibodies, including rabbit anti-GFAP (1:250, Abcam, USA), rabbit anti-Iba-1 (1:500, Abcam, USA), mouse anti-CD206 (1:50, Santa Cruz Biotechnology, USA), mouse anti-CD86 (1:100, Santa Cruz Biotechnology, USA), rabbit anti-C3 (1:200, MyBioSource, USA), and mouse anti-S100a10 (1:200, Invitrogen, USA). Following 2 h of incubation with the abovementioned fluorescence-labeled secondary antibody, images were acquired with a fluorescence microscope, and the MFI was determined.

### Imaging of Labeled Platelets

Platelets were isolated as described in Sect. Isolation of rat platelets, labeled in vitro with rhodamine-6 g (50 µL, 0.05%; Xinjiayuan Chemical Technology, China), and injected intravenously via the tail vein into sham rats or rats subjected to SAD. Twenty-four hours after injection, the rats were anesthetized with sodium pentobarbital (30 mg/kg), and 250 ml of PBS was perfused through the left ventricle to flush out the blood from the right atrium. After perfusion, the brain was excised for imaging as described in "Immunofluorescence".

### Western Blot Analysis of Signaling Proteins

Proteins extracted from microglia, astrocytes and brain tissues (after perfusion as described in Sect. Imaging of labeled platelets) were processed and subjected to immunoblotting using the Simple Wes System (Protein Simple, USA) and Size Separation Master Kit with Split Buffer (12–230 kDa) according to the manufacturer’s instructions. The following primary antibodies were used: rabbit anti-nuclear factor kappa B inhibitor alpha (1:30; IκBα; Abcam), rabbit anti-nuclear factor kappa B P65 (1:10; P65-NFκB; Abcam), rabbit anti-pP65-NFκB (1:20; Invitrogen), rabbit anti-CD41 (1:20; Abcam), mouse anti-β-actin (1:50; Abcam), and rabbit anti-Lamin B1 (1:50; Abcam). The immunoblots were analyzed using Compass software 4.0.0 (Protein Simple).

### Statistical Analysis

The data are expressed as the mean ± SEM. Pairwise comparisons were carried out using Student’s *t* test, while comparisons of three or more groups were carried out using one-way analysis of variance (ANOVA) with Tukey’s post hoc analysis. Statistical significance was defined as a two-tailed P < 0.05. All analyses were performed using GraphPad Prism 8.4.0 software (San Diego, CA, USA).

## Results

### ABR Dysfunction Induces Neuroinflammation

**Fig. 1:**
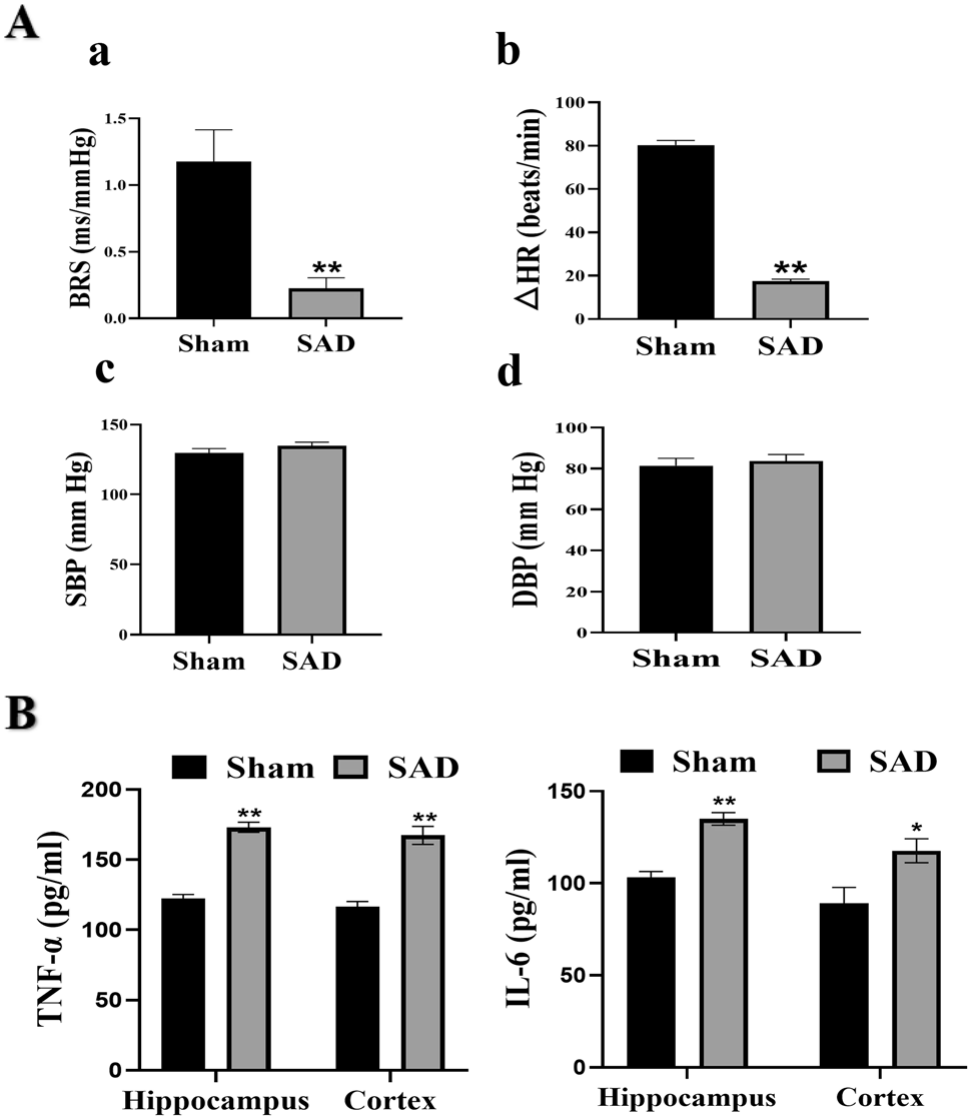
ABR dysfunction induces neuroinflammation. Animals were subjected to SAD or sham surgery. **A** BRS (**a**), the change in heart rate (∆HR, **b**), SBP (**c**) and DBP (**d**) were measured in conscious, free-moving rats. **B** Levels of TNF-α and IL-6 in hippocampus and cortex. The data are presented as the mean ± SEM (n = 6 animals per group). *P < 0.05, **P < 0.01 versus the sham group

After four weeks of SAD, rats developed ABR dysfunction, as evidenced by a considerable decrease in BRS (Fig. 1A, a; P < 0.01) without significant changes in SBP or DBP (Fig. 1A, c, d; P > 0.05) and a decrease in heart rate < 20 beats/min (Fig. 1A, b; P < 0.01) when SBP was raised 50 mmHg. To investigate whether ABR dysfunction elicits neuroinflammation, we assessed the levels of inflammatory cytokines in the hippocampus and cortex. Compared to sham rats, rats subjected to SAD had significantly higher levels of the proinflammatory cytokines TNF-α and IL-6 in both the hippocampus and cortex (Fig. 1B, P < 0.05), suggesting that ABR dysfunction induces neuroinflammation.

### ABR Dysfunction Causes Glial Activation

**Fig. 2:**
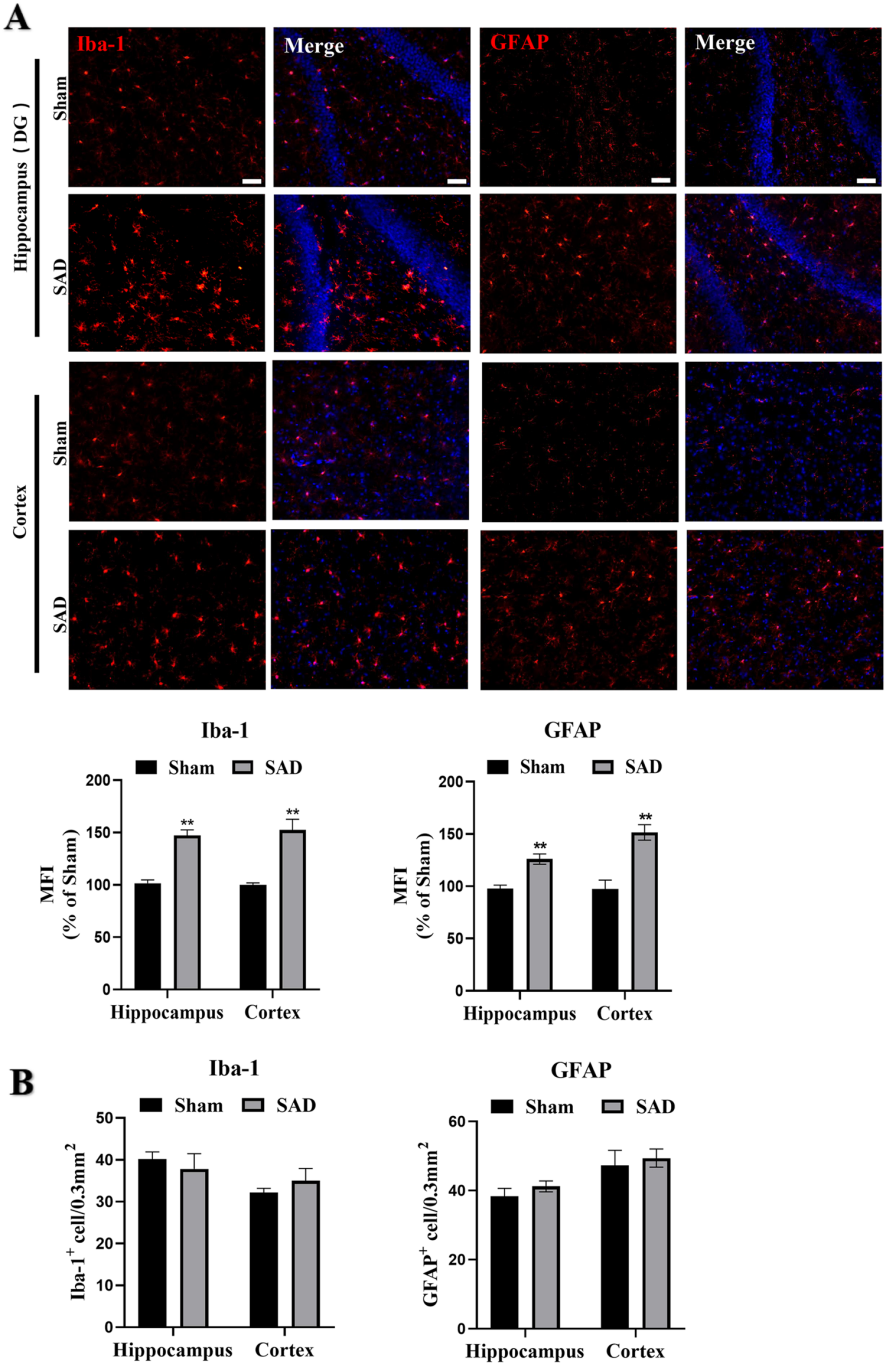
**A**BR dysfunction induces glial activation. Animals were subjected to SAD or sham surgery. **A** Representative immunofluorescence images and quantitative analysis of the protein levels of Iba-1, a marker of microglia, and GFAP, a marker of astrocytes, in the hippocampus (DG) and cortex. MFI indicates mean fluorescence intensity. **B** Quantitative analysis of the numbers of Iba-1 + microglia and GFAP + astrocytes in the hippocampus (DG) and cortex. The scale bars indicate 50 μm. The data are presented as the mean ± SEM (n = 6 animals per group). *P < 0.05, **P < 0.01 versus the sham group

**Fig. 3:**
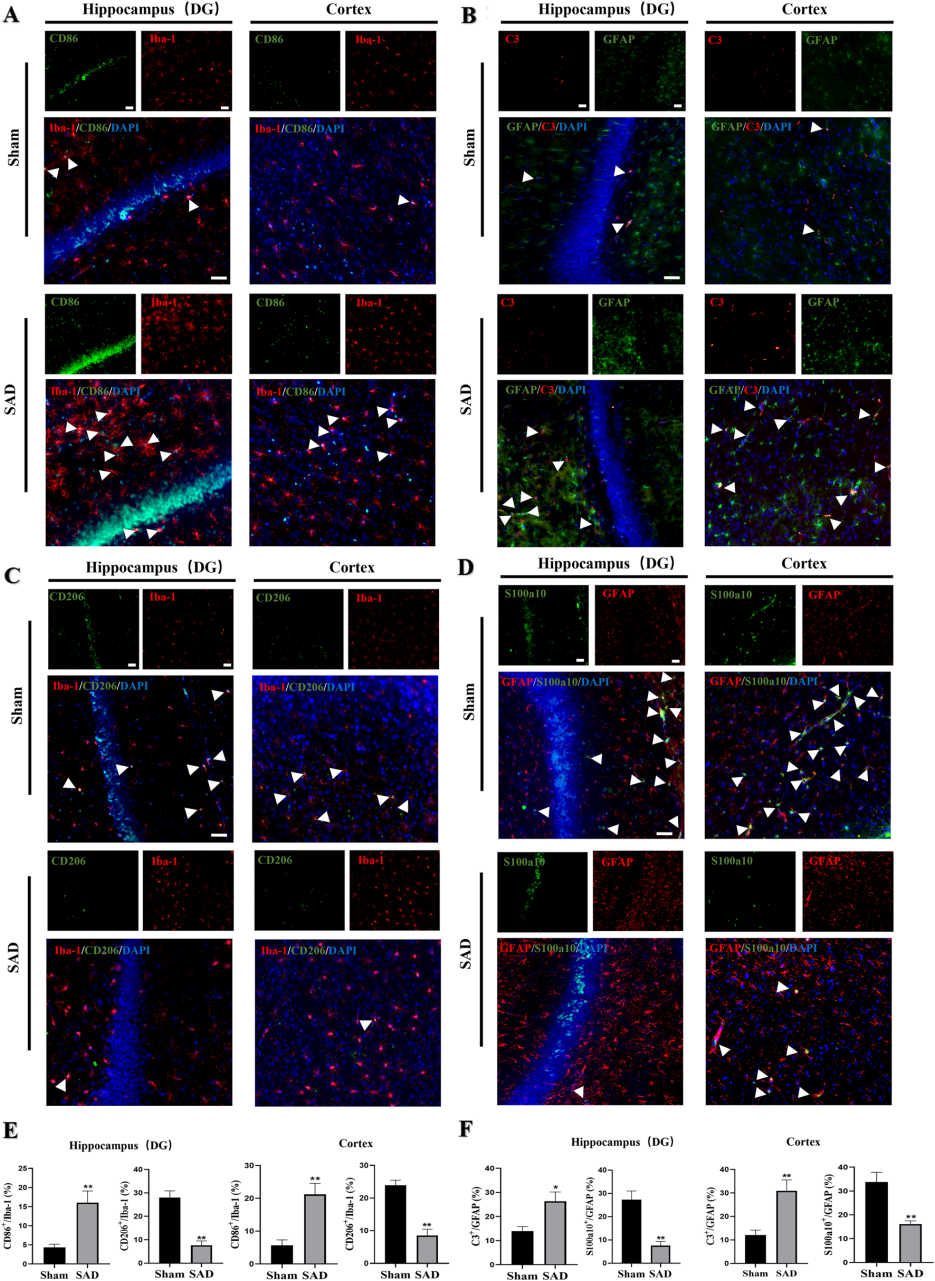
ABR dysfunction induces phenotypic polarization of glia. Animals were subjected to SAD or sham surgery. **A** and **C **Representative immunofluorescence images of CD86 (**A**) and CD206 (**C**) protein expression of Iba-1-positive cells in the hippocampal DG and cortex. The white arrow indicates a Iba-1 cells (red) stained for CD86 or CD206 (green) and with DAPI (blue). **B** Representative immunofluorescence images of C3 protein expression on GFAP-positive cells in the hippocampus (DG) and cortex. The white arrow indicates a GFAP cells (green) stained for C3 (red) and with DAPI (blue). **D** Representative immunofluorescence images of S100a10 protein expression on GFAP-positive cells in the hippocampus (DG) and cortex. The white arrow indicates a GFAP cells (red) stained for S100a10 (green) and with DAPI (blue). The scale bars indicate 50 μm. **E** and **F **Quantification of the ratio of CD86 and CD206 in Iba-1-positive cells (**E**) and the ratio of C3 and S100a10 in GFAP positive cells (**F**) in the hippocampal DG and cortex. The data are presented as the mean ± SEM (n = 6 animals per group). ^*^P < 0.05, ^**^P < 0.01 versus the sham group

Since phenotypic polarization of glial cells is implicated in the release of cerebral inflammatory cytokines [28, 34], we next investigated the levels of Iba-1 as a marker of microgliosis and GFAP as a marker of astrogliosis. The expression of both markers in the hippocampus and cortex was significantly higher in rats subjected to SAD than in sham animals (Fig. 2A, P < 0.01), indicating activation of cerebral microglia and astrocytes in rats with ABR dysfunction. However, there was no significant difference in the numbers of Iba-1-positive cells and GFAP-positive cells in the hippocampus and cortex between sham rats and rats subjected to SAD (Fig. 2B, P > 0.05). Because microglia and astrocytes can be polarized toward a proinflammatory phenotype (M1 and A1, respectively) or an anti-inflammatory phenotype (M2 and A2) [26], we assessed the numbers of microglia and astrocytes of each phenotype. We defined M1 microglia as those positive for both CD86 (green) and Iba-1 (red) and M2 microglia as those positive for both CD206 (green) and Iba-1 (red). Rats subjected to SAD showed more M1 microglia and fewer M2 microglia in the hippocampus and cortex than sham animals (Fig. 3A, C, E; P < 0.01). Similarly, rats subjected to SAD showed more A1 astrocytes, which were double labeled for C3 (red) and GFAP (green), and fewer A2 astrocytes, which were double positive for S100a10 (green) and GFAP (red) (Fig. 3B, D, F; P < 0.05, P < 0.01). The results suggest that ABR dysfunction induces the activation of M1 microglia and A1 astrocytes.

### ABR Dysfunction Leads to the Activation of Platelets and Induces Platelets to Enter and Accumulate in the Brain

**Fig. 4:**
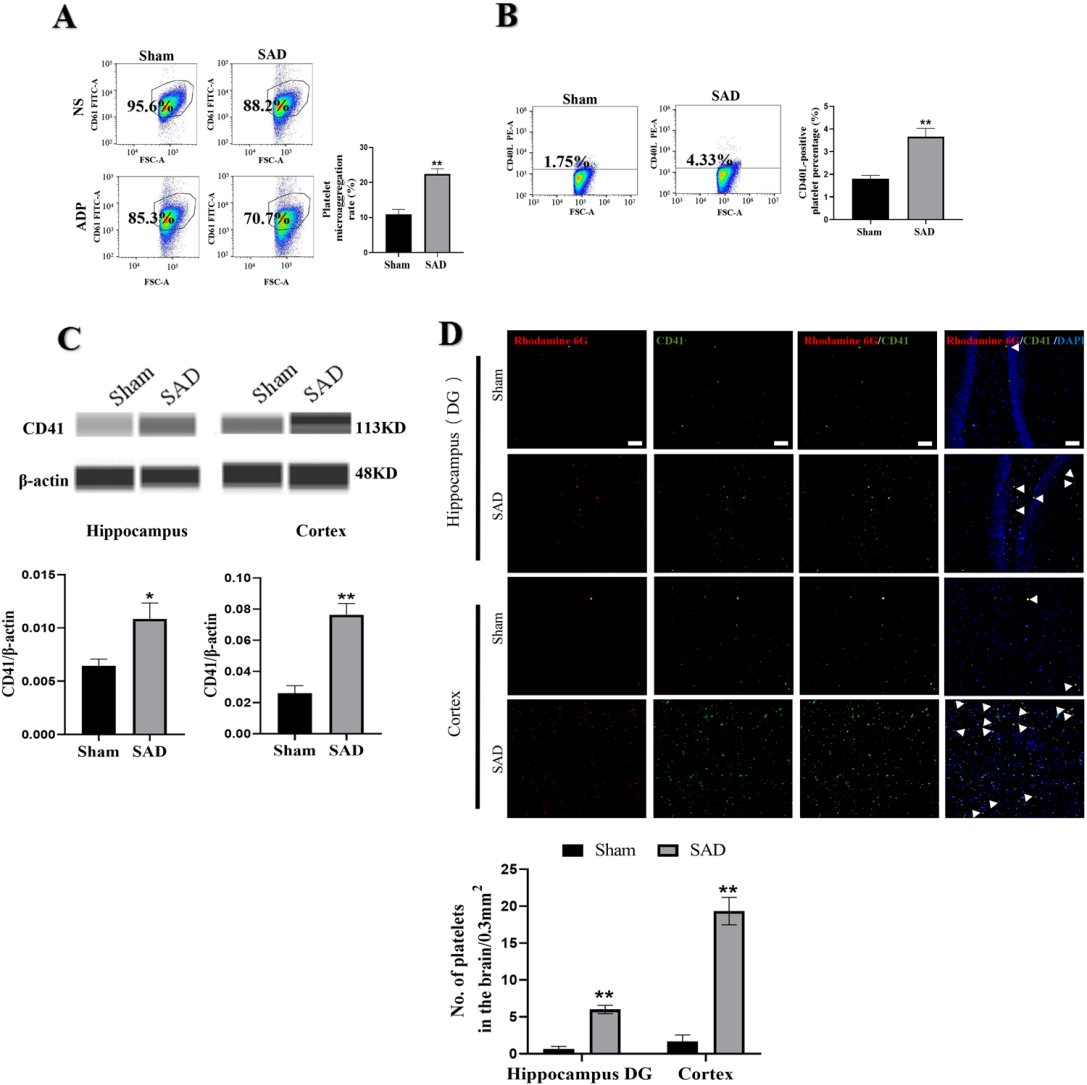
ABR dysfunction leads to platelet activation and induces platelets to enter and accumulate in the brain. Animals were subjected to SAD or sham surgery. **A** Flow cytometry analysis of the platelet microaggregation rate. **B** Flow cytometry analysis of the percentage of CD40L + platelets. **C** Representative Western blot images and quantification of CD41 protein levels in the hippocampi and cortices of rats after the blood was thoroughly flushed from the vessels with PBS. **D** Representative immunofluorescence images and quantitative analysis of the number of platelets deposited in the hippocampal DG and cortex. Twenty-four hours after intravenous injection of rhodamine-labeled platelets, the blood was thoroughly flushed from the vessels, and rat brain tissues were immediately subjected to immunofluorescence staining for CD41. The white arrow indicates a rhodamine-labeled platelet (red) stained for CD41 (green). The scale bars indicate 50 μm. The data are presented as the mean ± SEM (n = 6 animals per group). ^*^P < 0.05, ^**^P < 0.01 versus the sham group

Given the role of platelets in inflammation, we assessed whether ABR dysfunction is associated with platelet activation. Indeed, we found that SAD animals showed higher platelet microaggregation and more CD40L-positive platelets than sham rats (Fig. 4A, B; P < 0.01).

To verify more directly that platelet activation drives neuroinflammation induced by ABR dysfunction, we initially evaluated whether the brains of rats subjected to SAD had a higher number of platelets. After the blood was thoroughly flushed from the vessels, the rat brain was removed. Western blotting showed that rats subjected to SAD had higher levels of the platelet marker CD41 in the hippocampus and cortex than sham rats (Fig. 4C, P < 0.05). Additionally, 24 h after intravenous injection of rhodamine-labeled platelets, the blood was thoroughly flushed from the vessels, and rat brain tissues were immediately subjected to immunofluorescence staining for CD41 (a platelet marker). We found that there were more platelets in the hippocampus and cortex in animals subjected to SAD than sham animals (Fig. 4D, P < 0.01). These results suggest that ABR dysfunction can induce platelets to enter and accumulate in the brain.

### Platelet CD40L from Rats with ABR Dysfunction Induces Activation of M1 Microglia/A1 Astrocytes

**Fig. 5:**
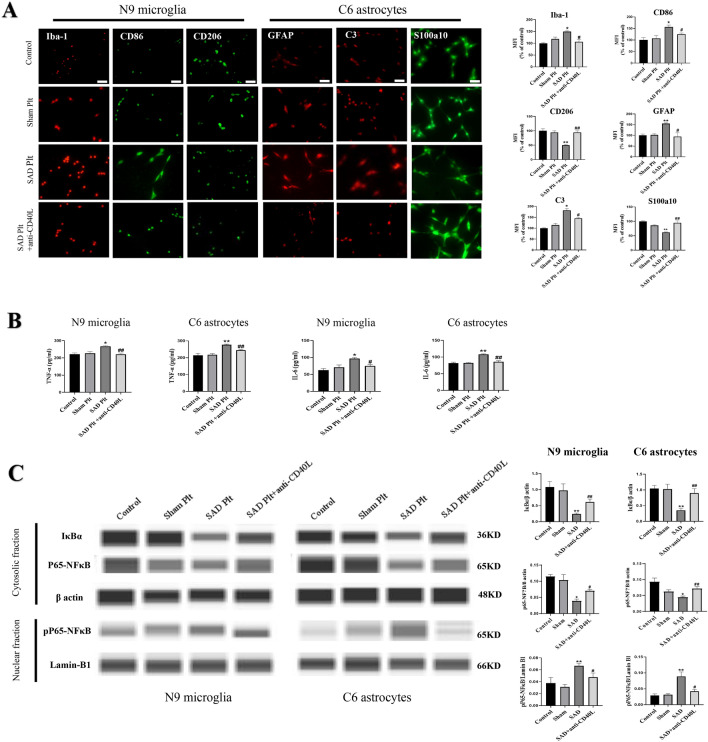
Platelet CD40L from rats with ABR dysfunction induces glial polarization in vitro. N9 microglia and C6 astrocytes were treated with platelets isolated from rats subjected to SAD or sham surgery. **A** Representative fluorescence images and quantitative analysis of the numbers of M1 microglia (CD86+/Iba-1+), M2 microglia (CD206+/Iba-1+), A1 astrocytes (C3+/GFAP) and A2 astrocytes (S100a10+/GFAP) after treatment with platelets or platelets plus an anti-CD40L antibody. The scale bars indicate 40 μm. MFI indicates mean fluorescence intensity. **B** Quantification of the release of TNF-α and IL-6 from N9 microglia and C6 astrocytes. **C** Representative Western blot images and quantification of cytoplasmic IκBα and NFκB-p65 and nuclear NFκB-pP65 levels in N9 microglia and C6 astrocytes cultured with platelets. Control, sham Plt, SAD Plt, and SAD Plt + anti-CD40L indicate glial cells cultured with PBS, glial cells cultured with platelets isolated from sham rats, glial cells cultured with platelets isolated from rats subjected to SAD, and glial cells cultured with CD40L antibody-pretreated platelets isolated from rats subjected to SAD, respectively. The data are presented as the mean ± SEM (n = 3–4 per group). ^*^P < 0.05, ^**^P < 0.01 versus the sham group. ^#^P < 0.05, ^##^P < 0.01 versus the SAD group

Next, using in vitro cultures of N9 microglia and C6 astrocytes, we examined whether platelets from rats with ABR dysfunction can polarize glial cells toward a proinflammatory phenotype. Immunostaining showed that the levels of the microglial marker Iba-1, the M1 phenotype marker CD86, the astrocyte marker GFAP and the A1 phenotype marker C3 as well as the proinflammatory factors TNF-α and IL-6 were significantly increased in astrocytes and microglia incubated with platelets isolated from rats subjected to SAD compared with those incubated with platelets isolated from sham rats (Fig. 5A, B; P < 0.05). Furthermore, the levels of the M2 phenotype marker CD206 and the A2 phenotype marker S100a10 were decreased (Fig. 5A, B; P < 0.05).

To further evaluate the involvement of platelet CD40L from rats with ABR dysfunction in the determination of glial phenotype, microglia and astrocytes were incubated with platelets isolated from rats subjected to SAD after being treated with a CD40L neutralizing antibody (30 µg/mL). The results revealed that the polarization of microglia and astrocytes toward a neurotoxic phenotype and the release of proinflammatory cytokines from both microglia and astrocytes were blocked (Fig. 5A, B; P < 0.05), suggesting that ABR dysfunction may proinflammatory microglial and astrocyte polarization through platelet CD40L.

We asked whether CD40L signaling in platelets may activate NFκB signaling in microglia and astrocytes, given that such signaling is overactivated in gliosis [35]. To explore the effects of platelet CD40L isolated from rats with ABR dysfunction on glial cell polarization, changes in NFκB signaling were analyzed by Western blotting. The levels of IκBα and P65-NFκB in the cytoplasmic fraction were significantly reduced while the level of pP65-NFκB in the nuclear fraction was increased in N9 microglia and C6 astrocytes incubated with platelets isolated from rats subjected to SAD compared with those isolated from sham rats (Fig. 5C, P < 0.05). These changes were blocked by pretreating the platelets isolated from rats subjected to SAD with an anti-CD40L antibody (30 µg/mL) (Fig. 5C, P < 0.05). These results suggest that during ABR dysfunction, platelet CD40L acts via NFκB signaling to activate proinflammatory glia.

### Platelet Inhibition Decreases Platelet Deposition in the Brains of Rats with ABR Dysfunction

**Fig. 6:**
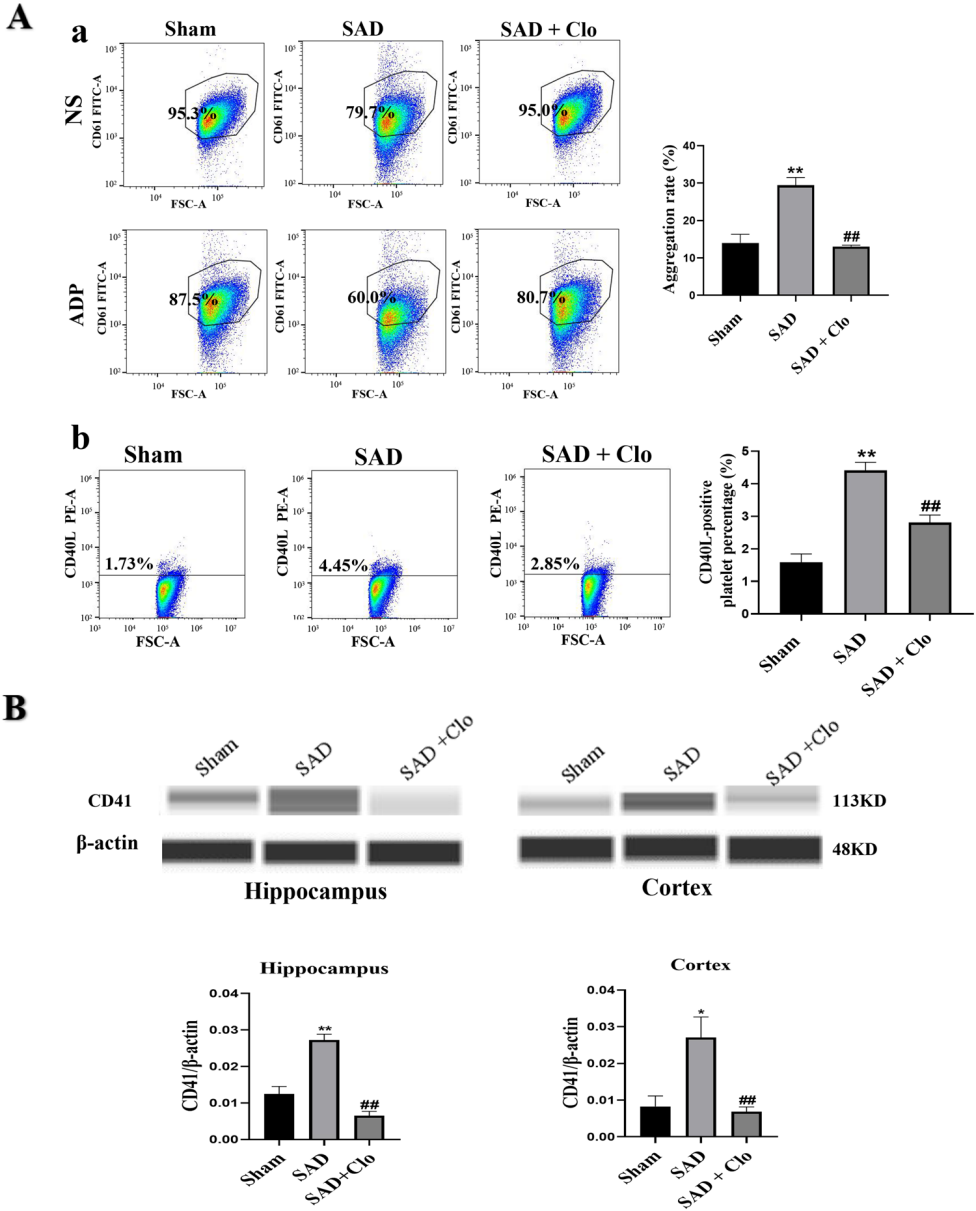
Platelet inhibition decreases platelet activation and platelet deposition in the brains of rats with ABR dysfunction. Animals were subjected to SAD or sham surgery. Clopidogrel was administered by gavage for five for 5 weeks. **A** Flow cytometry analysis of the platelet microaggregation rate (a) and percentage of CD40L + platelets (b) in sham rats and rats subjected to SAD. **B** Representative Western blot images and quantification of CD41 protein expression in hippocampus and cortex. The data are presented as the mean ± SEM (n = 6 animals per group). ^*^P < 0.05, ^**^P < 0.01 versus the sham group. ^#^P < 0.05, ^##^P < 0.01 versus the SAD group

Initially, we assessed the inhibitory effect of clopidogrel on platelet activation during ABR dysfunction. After five weeks of treatment with clopidogrel, flow cytometry showed that platelet activation in rats subjected to SAD was attenuated, as evidenced by weaker platelet microaggregation (Fig. 6A, a; P < 0.01) and a decrease in the percentage of CD40L-positive platelets (Fig. 6A, b; P < 0.01).

Then, the protein level of CD41 in the hippocampus and cortex was measured by Western blotting to evaluate platelet deposition in the brain. CD41 expression in the hippocampus and cortex was reduced in clopidogrel-treated rats subjected to SAD compared with PBS-treated rats subjected to SAD (Fig. 6B, P < 0.01). These results indicate that clopidogrel significantly inhibits platelet activation and decreases platelet deposition in the hippocampi and cortices of rats with ABR dysfunction.

### Inhibiting Platelet Activity Alleviates Neuroinflammation and Activation of A1 Astrocytes and M1 Microglia During ABR Dysfunction

**Fig. 7:**
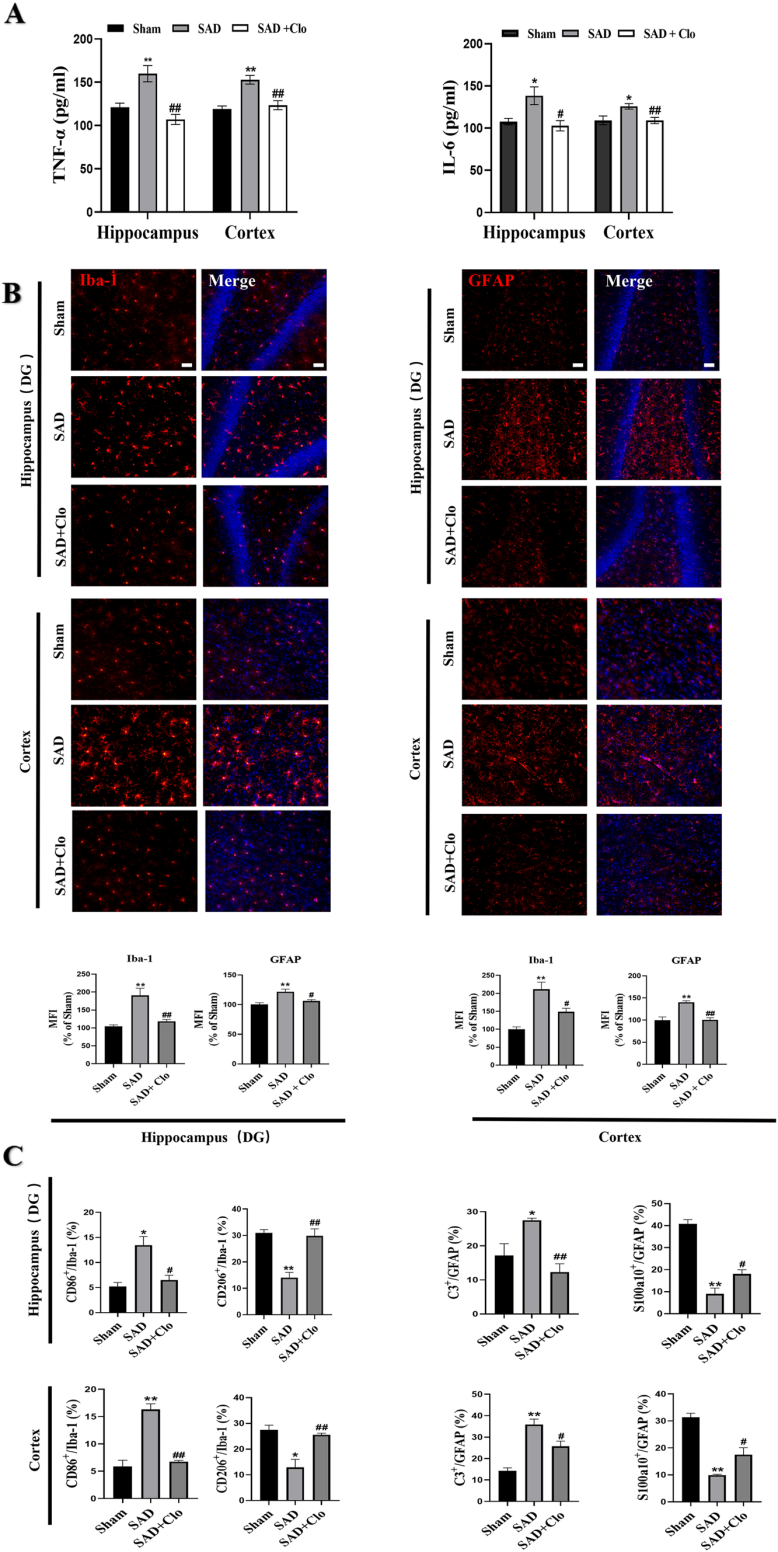
Inhibition of platelets following ABR dysfunction attenuates neuroinflammation and activation of M1 microglia and A1 astrocytes in vivo. Animals were subjected to SAD or sham surgery. Clopidogrel was administered by gavage for five for 5 weeks. **A** Levels of TNF-α and IL-6 in rat hippocampus and cortex. **B** Representative immunofluorescence images and quantification of Iba-1 and GFAP protein expression in the hippocampal DG and cortex. MFI indicates mean fluorescence intensity. **C** Quantitative analysis of the numbers of M1 microglia (CD86+/Iba-1+), M2 microglia (CD206+/Iba-1+), A1 astrocytes (C3+/GFAP+) and A2 astrocytes (S100a10+/GFAP+) in the hippocampal DG and cortex. The data are presented as the mean ± SEM (n = 6 animals per group). ^*^P < 0.05, ^**^P < 0.01 versus the sham group. ^#^P < 0.05, ^##^P < 0.01 versus the SAD group

After treatment with clopidogrel for 5 weeks, rats subjected to SAD showed reduced neuroinflammation, as revealed by decreased levels of inflammatory cytokines such as TNF-α and IL-6 in the hippocampus and cortex (Fig. 7A, P < 0.05). Clopidogrel inhibited the expression of two glial cell markers in the brain, suggesting that it inhibited glial cell activation (Fig. 7B, P < 0.05). Moreover, clopidogrel induced an increase in the numbers of proinflammatory M1 microglia and A1 astrocytes and a concomitant decrease in the number of anti-inflammatory cells in the hippocampi and cortices of rats subjected to SAD, as reflected by decreases in the numbers of CD86+/Iba-1 + and C3+/GFAP + cells (Fig. 7C, P < 0.05) and increases in the numbers of CD206+/Iba-1 + and S100a10+/GFAP + cells (Fig. 7C, P < 0.05). These results indicate that inhibiting platelet activity can alleviate neuroinflammation and activation of A1 astrocytes and M1 microglia during ABR dysfunction.

### Inhibition of Platelets From Rats with ABR Dysfunction in Vitro attenuates Activation of A1 Astrocytes and M1 Microglia

**Fig. 8:**
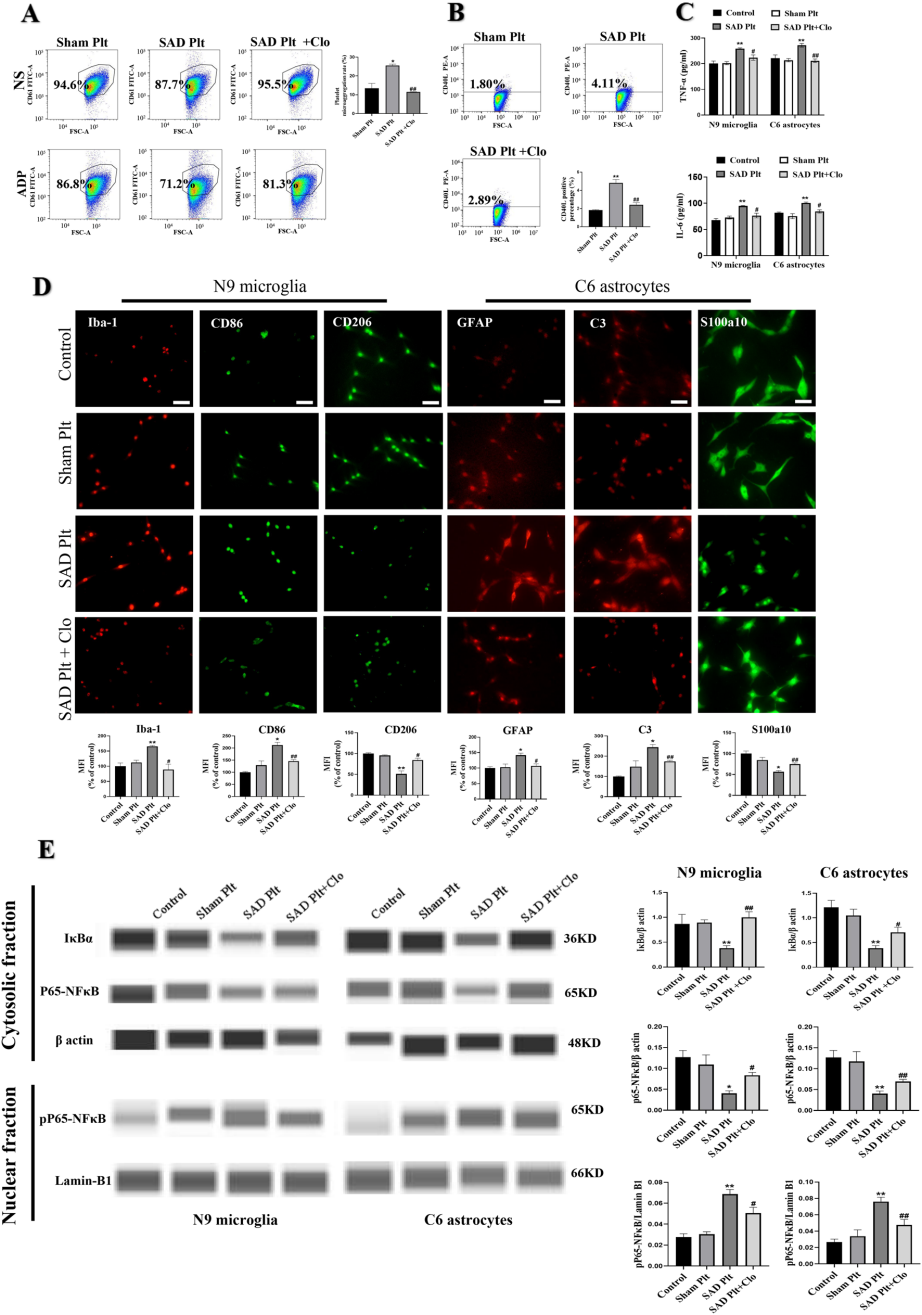
In vitro inhibition of platelets blocks the ability of ABR dysfunction to promote M1 polarization of microglia and A1 polarization of astrocytes. After platelets were isolated from rats subjected to SAD or sham surgery, they were treated with clopidogrel (Clo) or PBS. Then, these platelets were cultured with N9 microglia and C6 astrocytes. **A** Flow cytometry analysis of the platelet microaggregation rate. **B** Flow cytometry analysis of the percentage of CD40L + platelets. **C** Release of TNF-α and IL-6 from cultured N9 microglia and C6 astrocytes. **D** Representative fluorescence images and quantitative analysis of the numbers of M1 microglia (CD86+/Iba-1+), M2 microglia (CD206+/Iba-1+), A1 astrocytes (C3+/GFAP) and A2 astrocytes (S100a10+/GFAP). **E** Representative immunoblots and quantification of cytoplasmic IκBα and NFκB-p65 levels as well as nuclear NFκB-pP65 levels in N9 microglia and C6 astrocytes. Sham Plt, SAD Plt, and SAD Plt + Clo indicate PBS-treated platelets isolated from sham rats, PBS-treated platelets isolated from rats subjected to SAD, and clopidogrel-treated platelets isolated from rats subjected to SAD, respectively (A, B). Control, sham Plt, SAD Plt, and SAD Plt + Clo indicate glial cells cultured with PBS, glial cells cultured with platelets isolated from sham rats, glial cells cultured with platelets isolated from rats subjected to SAD, and glial cells cultured with clopidogrel-pretreated platelets isolated from rats subjected to SAD, respectively (C–E). The data are presented as the mean ± SEM (n = 3–4 per group). ^*^P < 0.05, ^**^P < 0.01 versus the sham group. ^#^P < 0.05, ^##^P < 0.01 versus the SAD group

To further investigate the inhibitory effect of platelets on the activation of A1 astrocytes and M1 microglia, in vitro experiments were performed. Platelets were treated with clopidogrel, and we found that clopidogrel could significantly inhibit the increases in microaggregation and CD40L expression on the surface of platelets isolated from rats subjected to SAD (Fig. 8A, B; P < 0.01), suggesting that clopidogrel directly inhibits platelets from rats with ABR dysfunction.

Next, we investigated the impact of clopidogrel-mediated platelet inhibition on glial activation. We observed that compared to PBS-pretreated platelets from rats subjected to SAD, clopidogrel-pretreated platelets from rats subjected to SAD inhibited N9 microglial and C6 astrocyte activation, as indicated by decreased secretion of TNF-α and IL-6 from microglia and astrocytes (Fig. 8C, P < 0.05), reduced polarization toward the proinflammatory M1 and A1 phenotypes (Fig. 8D, P < 0.05) and increased polarization toward the anti-inflammatory M2 and A2 phenotypes (Fig. 8D, P < 0.05). Furthermore, we found that clopidogrel pretreatment significantly prevented IκBα degradation (Fig. 8E, P < 0.05) and NFкB nuclear translocation (Fig. 8E, P < 0.05) in microglia and astrocytes treated with platelets from rats with ABR dysfunction.

## Discussion

Using a rat model of ABR dysfunction and cultured microglia and astrocytes, we provide evidence that during ABR dysfunction, platelet activation promotes the proinflammatory polarization of microglia and astrocytes by signaling mediated by CD40L on the platelet surface and by NFκB signaling within glia. These proinflammatory glia then drive neuroinflammation. We further show that it may be possible to alleviate this neuroinflammation by inhibiting CD40L signaling or platelet activation.

It is well known that the ABR plays an important role in maintaining normal BP and heart rate [36]. When the ABR is disrupted by SAD, the rat heart beats faster in response to an increase in BP [37]. In our experiment, 4 weeks after SAD, rats presented a decrease in BRS and a change in heart rate of ≤ 20/min when SBP was increased by 50 mmHg. These results demonstrate that SAD can be used to successfully model ABR dysfunction in animals, which is consistent with previous studies [13, 38, 39].

Neuroinflammation is the immune response of the CNS to various injuries or stimuli, including stress, infection, tissue damage and autoimmune conditions [40, 41]. Long-term or excessive neuroinflammation, which represents the most common feature of various neurological diseases, causes neuronal damage, resulting in disorders such as neurodegenerative diseases, depression, pain, and stroke [42]. The important regulators of neuroinflammatory responses in the CNS are microglia and astrocytes [25]. After activation, the microglial and astrocyte populations become heterogeneous, with microglia and astrocytes being traditionally classified as neurotoxic (M1 microglia and A1 astrocytes) or neuroprotective (M2 microglia and A2 astrocytes) [43, 44]. One of the markers of neuroinflammation is the release of proinflammatory cytokines by glial cells in the brain [25]. In our study, we observed significant increases in TNF-α and IL-6 levels as well as the activation of M1 microglia and A1 astrocytes in the brains of rats subjected to SAD, demonstrating the occurrence of neuroinflammation induced by ABR dysfunction. Our data support previous reports by Liu et al. [10].

Based on the high expression of CD40L on the activated platelet surface, the existence of CD40 (the CD40L ligand) on the surface of microglia and astrocytes, and the role of platelets in inflammation [45, 46], we speculate that during ABR dysfunction, platelet CD40L might trigger neuroinflammation through the activation of microglia and astrocytes. The following evidence from this study supports our speculation: First, rats subjected to SAD exhibited higher expression of CD40L on the platelet surface and a higher level of platelet microaggregation; after intravenous injection of rhodamine-labeled platelets, more platelets were found to enter and accumulate in the hippocampus and cortex in rats subjected to SAD; moreover, there were more platelets in the brains of rats subjected to SAD. These results reveal that ABR dysfunction increases the expression of CD40L on the platelet surface and induces platelets to enter and accumulate in the brain. Second, when microglia and astrocytes were cultured with platelets from rats subjected to SAD in vitro, the numbers of proinflammatory M1 microglia and A1 astrocytes were increased and the numbers of anti-inflammatory M2 microglia and A2 astrocytes were decreased, which is consistent with the changes in rats subjected to SAD. All the above changes were blocked after platelets isolated from rats subjected to SAD were pretreated with an anti-CD40L antibody. These data suggest that ABR dysfunction induces neuroinflammation by activating microglia and astrocytes through CD40L on the platelet surface. Third, in vitro treatment with platelets isolated from rats subjected to SAD resulted in marked NFκB activation in microglia and astrocytes, which was diminished by pretreating the platelets from rats subjected to SAD with an anti-CD40L antibody, further indicating that platelet CD40L activates proinflammatory glia through NFκB signaling during ABR dysfunction. Taking into account all this evidence, we believe that ABR dysfunction promotes neuroinflammation by activating the platelet CD40L/NFκB signaling pathway in microglia and astrocytes.

Because ABR dysfunction is an important characteristic of hypertension, we suggest that platelet-induced glial cell activation and neuroinflammation in hypertension may [47], at least in part, be due to ABR dysfunction. Additionally, our study suggests that when treating patients with CNS disorders related to neuroinflammation and hypertension, attention should be paid to ABR dysfunction in addition to increased BP. Given that microglia have been shown to activate astrocytes in the CNS [48], microglia may play a role in astrocyte polarization during ABR dysfunction.

Although hypertension has been linked to platelet CD40L-mediated activation of microglia and astrocytes [47], whether platelets can enter the brain and contribute to neuroinflammation is unclear. We found here that subjecting rats to SAD caused peripheral blood platelets to enter the hippocampus and cortex, where they appeared to promote the polarization of microglia and astrocytes toward proinflammatory phenotypes. Our study may provide new insight into the mechanism by which platelets cause CNS inflammation. Because ABR dysfunction is associated with abnormal sympathetic nerve firing and levels of angiotensin II [49, 50], these neurohumoral abnormalities might be important causes of platelet activation during ABR dysfunction. Furthermore, SAD can increase BP variability in rats, so increased BP fluctuations concomitant with ABR decline may also cause platelet activation [51]. CD40L has been demonstrated to be expressed in platelets following platelet activation [52]. Thus, high expression of CD40L on the platelet surface may be related to platelet activation during ABR dysfunction. The molecular mechanisms underlying the upregulation of CD40L in platelets during ABR dysfunction may involve protein kinase C, internal Ca^2+^ and GP IIb/IIIa [52, 53], as well as the prostaglandin/cAMP and NO/cGMP pathways [54]. Further studies need to be carried out to determine how ABR dysfunction leads to the activation of platelets. In addition, other parameters related to platelets, such as soluble CD40L levels and mean platelet volume, should be further investigated.

In our study, we also assessed whether inhibiting platelet activation ameliorates neuroinflammation during ABR dysfunction. Clopidogrel is used as an antiplatelet agent for the treatment of hypertension [55], cerebral ischemia [56], and congestive heart failure [57]. In our study, we first confirmed that clopidogrel hampered CD40L expression on the platelet surface and platelet activation in rats subjected to SAD. Then, we found that clopidogrel significantly alleviated neuroinflammation, decreased the numbers of proinflammatory M1 microglia and A1 astrocytes, increased the numbers of anti-inflammatory M2 microglia and A2 astrocytes, and reduced platelet accumulation in the hippocampi and cortices of rats subjected to SAD. The beneficial effect of clopidogrel in vivo was similar to that *in vitro.* Through in vivo and in vitro studies of clopidogrel, we not only provide evidence that it inhibits platelets to alleviate neuroinflammation caused by ABR dysfunction but also provide further support for the involvement of platelet CD40L in neuroinflammation induced by ABR dysfunction. ABR dysfunction in our SAD animal model resulted from physical and chemical damage [58, 59], and complete SAD was achieved in our study; therefore, inhibition of platelet activation by clopidogrel in rats subjected to SAD did not seem to improve ABR function in the present study. However, further studies should address this issue.

Activation of the baroreflex is often used clinically to treat diseases caused by neuroinflammation. This typically involves the electrical stimulation of the aortic depressor nerve or treatment with low-dose ketanserin (0.1 mg/kg) [60, 61]. Our results support the use of antiplatelet drugs alone or combined with electrical stimulation or ketanserin to treat CNS inflammatory disorders associated with ABR dysfunction. Additionally, identifying more effective inhibitors of glial cell polarization may aid the treatment of neuroinflammation.

Taken together, our results demonstrate that during ABR dysfunction, platelet activation might promote the proinflammatory polarization of microglia and astrocytes by signaling mediated by CD40L on the platelet surface and by NFκB signaling within glia, resulting in neuroinflammation. Our results provide novel insight into the pathogenesis of ABR dysfunction-induced neuroinflammation and suggest that targeting platelet CD40L is beneficial for the treatment of CNS disorders associated with ABR dysfunction.

## Data Availability

All data generated or analyzed during this study are included in this paper.
